# Disrupted and Disconnected Post Disaster: Associations Between the Social and Built Environment and Loneliness During COVID-19 in a U.S. Gulf Coast Sample

**DOI:** 10.3390/ijerph22020203

**Published:** 2025-01-31

**Authors:** Samer Atshan, Lynsay Ayer, Andrew M. Parker, JoNell Strough, Bonnie Ghosh-Dastidar

**Affiliations:** 1 RAND Corporation, Santa Monica, CA 90401, USA; bonnieg@rand.org; 2 RAND Corporation, Arlington, VA 22202, USA; layer@rand.org; 3 RAND Corporation, Pittsburgh, PA 15213, USA; parker@rand.org; 4 Department of Psychology, West Virginia University, Morgantown, WV 26506, USA; jstrough@wvu.edu

**Keywords:** loneliness, COVID-19, social–built environment, routine disruptions, housing distress, community resilience

## Abstract

Loneliness, a significant public health issue, was exacerbated during the COVID-19 pandemic, particularly in disaster-prone regions like the U.S. Gulf Coast. This study examined how social and built environmental factors were associated with pandemic-related disruptions and loneliness among respondents from the third wave of the Survey of Trauma, Resilience, and Opportunity among Neighborhoods in the Gulf (STRONG). Using a retrospective measure of loneliness (pre-pandemic vs. during pandemic), we found that loneliness increased significantly during the pandemic. Using a measure of routine behavior disruptions and measures of both objective (e.g., parks, walkability, etc.) and subjective (e.g., neighborhood safety, social cohesion, etc.) environmental factors, we found that disruptions to daily routines strongly predicted higher loneliness, and subjective measures, such as neighborhood safety, social cohesion, and lacking post-disaster social support, were more salient predictors of loneliness than objective factors such as the number of parks in one’s neighborhood. Difficulty accessing green spaces and housing distress were linked to greater COVID-19 disruptions, indirectly contributing to loneliness. These findings highlight the importance of safe, supportive, and accessible social and physical environments in mitigating loneliness and enhancing community resilience during crises.

## 1. Introduction

Loneliness is the distressing experience associated with a cognitive discrepancy between perceived and desired social connection [1,2,3]. The health impacts of loneliness are profound and varied, including heightened risk of cardiovascular disease, weakened immune function, anxiety, depression, cognitive decline, and even premature mortality [4,5,6]. While loneliness is an inherently personal experience, it results from a complex interplay of factors across different levels of analysis (e.g., family/household membership, cultural/societal norms, and policies/legislation). In line with human ecological theory [7], loneliness can be construed as emerging within a social-ecological system. Social-ecological systems can be studied through several nested levels of interaction, ranging from the micro-system encompassing personal characteristics, to the meso- and macro- systems, which include a person’s neighborhood, community, and society [8]. Features of socio-ecological systems affect the incidence, development, and intensity of loneliness [9,10]. The social and built environment is one ecological factor that can impact loneliness. In fact, the first pillar in the U.S. Office of the Surgeon General’s strategy detailing policy responses to the growing epidemic of loneliness is strengthening social infrastructure in communities by designing the built environment in a way that promotes social connection [11]. Yet, investigations into how social and ecological (i.e., extra-individual) factors impact social connection are limited. This study aimed to explore how both objective (observable facets) and subjective features (perceptions) of the social and built environments were associated with experiences of disruption to routine behaviors and loneliness during COVID-19, a time when restrictions on social interactions were put in place.

### 1.1. Literature Review

Social and built environments encapsulate the ecosystem that shapes and is shaped by social interactions and physical infrastructure. “Social” refers to the socioeconomic and cultural dimensions of neighborhoods and communities, including crime, social support, collective efficacy, and social capital. “Built” refers to the physical attributes and amenities (or lack thereof) of the places where we live, work, travel, and play [12,13,14]. These have been shown to impact health and well-being across the lifespan [15,16,17]. The intersection of the built environment and socio-spatial characteristics can impact loneliness by impeding or facilitating mobility, access to social spaces and social capital, a sense of self-efficacy and collective efficacy, and the formation of place-based attachment and social identity [18,19,20,21].

The COVID-19 pandemic worsened mental health [22,23,24,25], including disruptions to daily routines [26] and impacts on loneliness and social isolation [27,28,29]. While many studies have focused on the personal factors of outcomes during the COVID-19 pandemic, few studies focused on aspects of the social and built environment, and those studies were limited in scope. The social and built environment may have been especially important when COVID-19-related health and safety policies (e.g., school closures, social distancing, etc.) imposed sudden and meaningful limits on how space could be used for social interactions. For example, Bower et al. demonstrated how personal factors such as income and persistent mental health problems but also neighborhood characteristics (perceived belonging) and societal factors (e.g., lockdowns within different states), all contributed to loneliness [30]. Finucane et al. found that pandemic experiences depended on the available social and physical resources [31]. Specifically, walkability in urban segregated neighborhoods was a significant moderator of the association between COVID-19 closure experiences and poor sleep quality, while social isolation partially mediated the relationship between closure experiences and poor behavioral health, even when controlling for individual-level demographic characteristics. Some other studies also suggested that during the COVID-19 pandemic, built-environment and neighborhood conditions were associated with mental health problems, psychological distress, and loneliness in particular [32,33]. For example, unaffordable housing, absence of natural lighting, frequency of bothersome noise, and housing structural concerns were all associated with experiencing loneliness even after controlling for neighborhood belonging and other demographic variables [21]. This may be because the pandemic’s lockdown measures confined individuals to their homes for extended periods, transforming residences into multifunctional spaces for work, education, and leisure. This sudden shift may have blurred the boundaries between personal and professional life, disrupting established daily routines. Neighborhoods and green spaces may have provided important social and physical spaces for individuals to connect with others safely to mitigate loneliness [34,35]. These spaces may have provided buffers that mitigated disruption to daily routines experienced during COVID-19. The range of studies in this area shows that social and built environments could have affected loneliness during COVID-19 through a multitude of pathways, yet each study tends to focus on one or two aspects of the social and built environment. What has not been thoroughly explored, however, is how different features of the social and built environments *jointly* affected experiences of disruption to routine behaviors and experiences of loneliness during the pandemic.

### 1.2. Context of This Study

This study aims to explore how different aspects of the social and built environment were associated with COVID-19 experiences of disruption to routine behaviors and loneliness during the pandemic among residents across the adult lifespan within the U.S. Gulf Coast. This region, encompassing coastal counties in Alabama, Florida, Louisiana, Mississippi, and Texas, has faced numerous disasters over the last two decades that exacerbated health disparities and socio-economic challenges [36,37,38,39,40]. The compounded effect of these adversities, including the pandemic, presents a unique opportunity to examine the nuanced interactions between disaster exposure, COVID-related disruptions (e.g., to work, social connection, etc.), and loneliness. This is salient in light of previous evidence of the impact of disasters on social support networks, community resilience, coping, and self-efficacy within this population [41,42,43,44].

**Figure 1:**
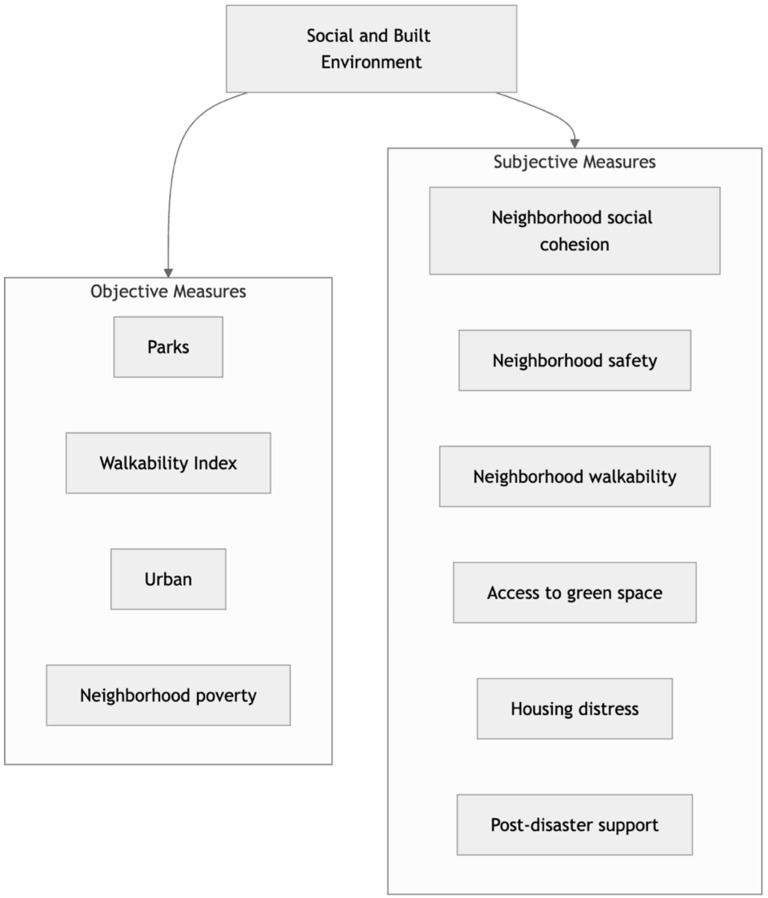
Social and built environment characteristics considered in this study.

In addition to assessing changes in loneliness during the pandemic, we focus on how the interplay between post-disaster social support and neighborhood characteristics such as safety and social cohesion as well as broader socio-spatial factors such as walkability and access to green spaces may have influenced disruptions from COVID-19 and loneliness during the pandemic. Neighborhood safety, neighborhood walkability, and being able to visit nearby parks during pandemic restrictions and work-from-home mandates may have provided a social space that facilitated safer face-to-face contact [20,34]. Neighborhoods with higher social cohesion may have allowed individuals to ask for and receive support through tough periods during the pandemic [35]. Furthermore, poor housing conditions and space constraints may have impeded individuals from creating the needed separation between their work life and home environments [45]. We therefore expected respondents who lived in neighborhoods with worse conditions (less safe, less green space, less walkable, less social cohesion, and poorer) to have experienced more disruption from COVID-19 and more loneliness. We also expected worse housing conditions to predict more disruption from COVID-19 and loneliness. To comprehensively capture the impact of the social and built environment, we considered both objective and subjective features of the environment, as shown in Figure 1. Objective characteristics included observable facets of the environment (e.g., number of parks within respondents’ area), while subjective characteristics included self-reported perceptions of respondents’ environment as collected from surveys. Including both types of measures allowed us to “ground” self-report measures with tangible and robust counterparts while also capturing how the subjective experience may be closer to psychological outcomes. This approach has been emphasized within several studies looking at relationships between the built environment and multiple health-related outcomes, including loneliness [20,21,46]. Very few of these studies, however, looked at disruption to routine behaviors as an important outcome during the pandemic or included a breadth of objective and subjective social and built environment characteristics to examine variance in loneliness. Moreover, even fewer considered changes in loneliness during the pandemic when compared to pre-pandemic times. These approaches provide a comprehensive picture of the protective or exacerbating roles those various elements of the social and built environment played within the Gulf Coast community during an unprecedented global health crisis.

Finally, the analysis considered important demographic factors that may have confounded the relationship between characteristics of the social and built environment on one hand and disruption from COVID-19 and loneliness on the other. A good example is socioeconomic status, which plays out at both the personal and neighborhood levels. Neighborhoods with worse socioeconomic conditions tend to have less access to green space [47]. At the same time, low individual socioeconomic status is its own risk factor for loneliness [48,49]. The analysis, therefore, included measures of both personal socioeconomic status and neighborhood socioeconomic conditions. In doing this, we addressed a common limitation of many analyses that draw on the relationship between loneliness and social and built environment characteristics [19,21], namely that personal and neighborhood socioeconomic variables may separately predict factors related to the social and built environment, which in turn might impact outcomes such as loneliness.

## 2. Methodology

### 2.1. Data

The primary data for this study came from the Survey of Trauma, Resilience, and Opportunity among Neighborhoods in the Gulf, a longitudinal study of residents living in U.S. Gulf Coast communities, focusing on disaster experiences and mental and behavioral health [50]. The first survey was fielded in 2016, six years after the Deepwater Horizon Spill, and aimed to understand the impacts of that disaster on a probability sample of 2520 adults [51]. Information about that survey’s sampling frame and response rates have been previously reported [42,52,53].

This analysis used data collected from the third wave of STRONG, fielded between May and August 2022. Survey questions focused on the effect of different aspects of COVID-19 on Gulf Coast residents’ health and well-being. However, two years into the COVID-19 pandemic, the weekly average of new U.S. COVID-19 infections at the end of May 2022 was at a high point—six times higher than it was in May 2021 (119,725 cases vs. 17,887 cases) [54]. We therefore expected COVID-19 to be a prominent issue, with respondents able to describe how it had impacted their lives up to that point. For this wave, 1907 surveys were mailed, and 542 online surveys were sent by email to respondents who had participated in the first wave in 2016, with both email and phone follow-up reminders, resulting in 598 completed surveys. The analytic sample in this study included 532 respondents who provided their addresses (to allow geographic matching to objective measures of the social and built environment) and completed at least half of the items on each scale of interest in this study.

### 2.2. Measures

#### 2.2.1. Loneliness

Loneliness levels were collected using the 3-item UCLA Loneliness Scale [55]. The scale asks respondents how often they lacked companionship, felt left out, and felt isolated from others with response options “hardly ever”, “some of the time”, and “often”. The scale was adapted to capture both pre-pandemic loneliness and loneliness during the pandemic by consecutively asking respondents about their feelings on a typical week before and after the start of the COVID-19 pandemic for each of the items. This resulted in six items, two for each item on the original scale (“In a typical week after/before the COVID-19 pandemic”). Pre-pandemic and pandemic loneliness measures were computed as sums of the three items referencing a typical week before the pandemic (α = 0.78) and a typical week after the start of the pandemic (α = 0.85). Scores ranged from 0 to 7, and higher scores indicated more loneliness.

#### 2.2.2. COVID-19 Disruption to Routine Behaviors

We measured the disruption COVID-19 may have caused respondents’ routine behaviors using a scale adapted by Parks et al. [56]. Respondents were asked to rate the extent to which, during the past 30 days, the COVID-19 outbreak prevented them from engaging in six routine behaviors: (1) interacting with friends, (2) taking care of usual daily chores, (3) doing usual job or other tasks, (4) being able to take care of family or other dependents, (5) planning for the future, and (6) imagining a return to “normal life” in the future. The response scale for all items was “not at all”, “a little”, “somewhat”, “very much”, and “totally”. Response options were coded from 0 to 4 and summed to create a disruption index ranging from 0 to 24, with higher values indicating more disruption due to COVID-19 (α = 0.88). In a study using a U.S. national sample, this scale showed similarly good internal consistency (α = 0.90) and was associated with psychological distress early during the pandemic [57].

#### 2.2.3. Objective Social and Built Environment

We included three objective measures of the social and built environment by merging geocoded survey responses (based on respondent addresses) with datasets that captured neighborhood characteristics at the smallest geographic resolution possible. First, we determined the number of parks in each respondent’s census tract (as of 2022) from the National Neighborhood Data Archive [58]. Second, we included the walkability score from the National Walkability Index for each respondent’s neighborhood, which was available at the census block group level. Using 2019 data, this measure is based on aspects of the built environment related to walking, such as street intersection density, proximity to transit stops, and diversity of land use based on measures of employment type within an area [59]. Third, whether respondents live in an urban or a rural area was determined based on the 2010 Rural-Urban Community Area (RUCA) Codes. RUCA codes are based on population density, urbanization, and tract-to-tract commuting flows. We defined urban areas as all census tracts that have 30% or more of their workers commuting to a Census Bureau-defined urbanized area (Categorization D) [60]. Finally, we included a neighborhood measure of socioeconomic status, the portion of the population living in poverty within each respondent’s census tract [61].

#### 2.2.4. Subjective Social and Built Environment

Six subjective measures of the social and built environment were included in this study: neighborhood social cohesion, neighborhood safety, neighborhood walkability, housing distress, access to green space, and post-disaster support.

- Neighborhood Social Cohesion: For perceptions of neighborhood social cohesion, five items drawn from The Los Angeles Family and Neighborhood Survey [62,63] were used to gauge respondents’ sense of social cohesion with others where they live: (1) people where I live are willing to help their neighbors, (2) where I live is close-knit, (3) people where I live can be trusted, (4) people where I live generally do not get along with each other, and (5) people where I live do not share the same values. Respondents rated their agreement with the statements on a 5-point Likert scale ranging from “strongly disagree” to “strongly agree”. Items 4 and 5 were reverse-coded, and all items were summed to create a 0-to-20 index, with higher values indicating higher social cohesion (α = 0.80);
- Neighborhood Safety: For perceptions of neighborhood safety, respondents rated their agreement with four statements using a 5-point Likert scale ranging from “strongly disagree” to “strongly agree”: (1) I feel safe walking around my neighborhood during the day, (2) I feel safe walking around my neighborhood during the evening, (3) my neighborhood is safe from crime, and (4) violence is a problem in my neighborhood. The last item was reverse-coded, and all items were summed to create a 0-to-16 index, with higher values indicating higher neighborhood safety (α = 0.83) [64,65];
- Neighborhood Walkability: To measure perceptions of walkability, we used the Neighborhood Environment Walkability Scale [66]. Respondents rated their agreement with three statements using a 5-point Likert scale ranging from “strongly disagree” to “strongly agree”: (1) there is so much traffic along nearby streets that it makes it difficult or unpleasant to walk in my neighborhood, (2) the speed on most streets near me is usually slow (30 mph or less), and (3) most drivers exceed the posted speed limits while driving in my neighborhood. The first and third items were reverse-coded, and all items were summed to create a 0-to-12 index, with higher values indicating greater perceived walkability (α = 0.43);
- Housing Distress: To measure structural problems people face within their homes, respondents were asked whether seven issues presented “a big problem”, “a small problem”, or “no problem at all” in their homes: (1) walls with peeling paint or broken plaster, (2) plumbing that does not work, (3) rats or mice, (4) cockroaches, (5) broken locks or no locks on the door to your unit, (6) broken windows or windows without screens, and (7) a heating system that does not work [67]. Items were summed into a 0-to-14 scale, with higher index indicated more housing distress (α = 0.78);
- Access to Green Space: Access to green space was measured using a single 5-point Likert item [65]: the parks or greenspaces closest to me are difficult to get to. Responses were reverse-coded, with higher scores indicated greater ease in accessing nearby green spaces;
- Post-Disaster Support: Respondents were asked if they had experienced impacts from the hurricanes (e.g., Ida, Zeta, and Sally) between 2019 and 2022 that had impacted communities along the Gulf Coast [68,69]. Respondents were asked which of these storms (if any) had impacted them most directly, with the follow-up question “Following the storm, how much help were you able to get from your community?*”* Response options were (1) “I did not need help”, (2) “all of the help needed”, (3) “most of the help needed”, (4) “very little of the help needed”, and (5) “no help”. A binary variable, indicating whether they lacked post-disaster support, was coded as “1” if respondents chose options (4) or (5) (i.e., little or no help) and “0” otherwise, which included if respondents did not experience any storm.

### 2.3. Analytic Approach

To assess pre-pandemic to pandemic changes in loneliness, an independent *t*-test compared average loneliness before and during the pandemic. To assess if objective and subjective measures of the social and built environment were associated with COVID-19 disruption and loneliness, multiple linear regressions were estimated in R v4.2.3 and RStudio v2023.12.0.369 for Mac (Rstudio, Boston, MA, USA). We first wanted to understand which factors were associated with routine disruption, so we first fit models that predicted COVID-19 disruption. We then fit models predicting loneliness using COVID-19 disruption and social–built environment factors to understand if both were associated with loneliness during the pandemic. For both COVID-19 disruption and loneliness, we fit multiple models that progressively added sets of predictors to allow for the examination of incremental variance explained by each set of variables, first starting with objective measures of the social and built environment, followed by subjective measures, and finally socio-demographic factors to control for confounding. Individual socio-demographic factors included gender, age, race, marital status, living alone, home ownership, employment, and income, which were observed to predict loneliness during the pandemic [70]. Finally, in the models predicting loneliness, we also included a model step that controls for pre-pandemic loneliness (measured retrospectively in 2022). Subject to assumptions [71], this step allowed us to understand how COVID-19 disruption and features of the social and built environment were associated with changes in loneliness from pre-pandemic to pandemic times.

### 2.4. Missing Data

We restricted the sample to respondents who provided their addresses, had non-missing responses on single items (e.g., access to green space), and had at least half of their scale items non-missing. Within this sample, remaining missing scale items and sociodemographic factors were imputed using predictive mean matching [72,73]. Imputation was carried out using multinomial logistic regression for race, proportional odds logistic regression for ordered categorical variables (age and income), and logistic regression for binary variables (gender, marital status, living alone, home tenure, and employment). Summary statistics and regression coefficients from each of the 500 imputed datasets were pooled using Rubin’s Rules to derive combined estimates and standard errors [74]. Multiple imputation was performed by chained equations using the *MICE* package in R [75].

## 3. Results

### 3.1. Sample Description

Table 1 describes the sociodemographic characteristics of the sample. Fifty-six percent of respondents were over age 65, and 61% of the sample was female. Most of the sample was White (80%), with Black respondents making up 18%, and those identifying as other races comprising 2.3%. Approximately 8% of the sample was Hispanic. Additionally, 56% of participants were married, and 4.6% reported living alone. The sample displayed a bimodal distribution in annual household income. Approximately 30% of respondents reported earning less than USD 30,000, while a similar proportion (36%) reported an income of USD 75,000 or more, indicating a significant variance in economic status within the sample. This income disparity was accompanied by a high rate of home ownership, with 81% of participants reporting owning their home. Only 39% of respondents indicated they were employed at the time of the study, which may be explained by the age of the sample.

Table 2 shows the characteristics of the sample on the main outcomes of interest (loneliness and COVID-19 disruption) and predictors representing various aspects of the social and built environment. The *t*-test comparing the sample on mean loneliness before and during the pandemic provided evidence that that on average, individuals were lonelier during the pandemic (t = 11.41, *p* < 0.001, CI = 1.01,1.44, df = 949). The mean score on the disruption index was 3.7, which is consistent with lower disruption levels. This is unsurprising in the later stages of the pandemic, as schools and businesses reopened, and in-person gatherings became more common despite elevated case rates. In comparison, the mean scores on the disruption index for a U.S. national sample of workers who were employed as of February 2020 was 9.2. This was based on a measure collected during the first summer after the start of the pandemic (June 2020) [57].

Based on objective measures of the social and built environment, 90% of the sample lived in urban neighborhoods. The mean poverty rate in census tracts where respondents lived was 14% and ranged from 0% to 61%. The average number of parks was 1.56, with census tracts having anywhere from 0 parks to 18 parks. The average walkability index in respondent census block groups was 8.1, which is below average by some standards [76].

Turning to subjective measures of the social and built environment, there was significant variability in respondents’ perceptions of their social and built environment, but multiple imputation did not substantively alter the distributions of these scores since the number of imputed observations ranged from 1 observation to at most 12 observations for some variables. Importantly, 18% of the sample reported that they lacked support after being affected by a recent storm. This was unsurprising given that in the second wave of the STRONG, around 44% of the sample indicated that they were adversely impacted by hurricanes that landed on the Gulf Coast between 2017 and 2018, such as Hurricane Harvey [44].

### 3.2. Correlations

Table 3 shows bivariate associations among outcomes and predictors of interest. Loneliness during the pandemic was highly correlated with pre-pandemic loneliness and COVID-19 disruption. Both loneliness during the pandemic and COVID-19 disruption were correlated with the subjective but not objective aspects of the social and built environment. Interestingly, objective measures did not always correlate with subjective measures. For example, perceptions of neighborhood walkability were mildly associated with the count of parks (r = 0.11, *p* < 0.01) but were uncorrelated with the census block group walkability index (r = 0.02, *p* > 0.05). Ease of access to green space was associated with higher perceptions of walkability but was not associated with the walkability index either. The walkability index was positively associated with neighborhood poverty rate, urban census tract status, and count of parks but was negatively associated with neighborhood safety. This suggests that areas with a higher walkability index were more urban than suburban or rural, consistent with expectations.

### 3.3. Regression Analysis

Table 4 shows the results of three multivariate regression analyses with experiencing COVID-19’s disruption to routine behaviors as the outcome. First was an analysis that used objective measures of the social and built environment as predictors (Model 1), followed by an analysis that used both objective and subjective measures (Model 2). Model 3 included adjustments for confounding variables (sociodemographic characteristics). Neighborhood poverty rate was the only objective measure associated with experiencing COVID-19 disruption; the effect was not statistically significant once we accounted for subjective measures in Models 2 and 3. Of the subjective measures, ease of access to green space was significantly associated with less disruption even after controlling for demographic covariates (B = −0.53, 95% CI = −0.91, −0.16); experiencing more housing distress was associated with experiencing more disruption (B = 0.39, 95% CI = 0.17, 0.60). There was a more modest association with the neighborhood social cohesion scale, where a one-point increase was associated with a 0.13 decrease on the disruption scale while holding other variables constant (95% CI = −0.26, 0.00).

Table 5 shows the results of multivariate regression analysis predicting loneliness during the COVID-19 pandemic. Similar to disruption, we first fit a model that only used objective measures as predictors, followed by a model that used both objective and subjective measures, and then adjusted for sociodemographic covariates. Objective measures of the social and built environment were not associated with loneliness during the pandemic (Model 4). Among subjective measures (entered in Model 5), a one-point increase on the neighborhood safety scale was associated with a 0.07 decrease on the loneliness scale (95% CI = −0.13, −0.01), and that effect was unchanged when accounting for disruption to routine behaviors (Model 6). Housing distress was associated with experiencing more loneliness, as seen in Model 5 (B = 0.13, 95% CI = 0.03, 0.22), but the effect was no longer significant once we accounted for disruption to routine behaviors (Model 6). Models 7 and 8 further controlled for demographic covariates and pre-pandemic loneliness. As seen in these models, lack of support following prior disasters (e.g., hurricanes) was associated with higher loneliness during the pandemic even when accounting for pre-pandemic loneliness and other sociodemographic covariates. Respondents who lacked support following prior disasters on average had a 0.41 (95% CI = 0.02, 0.79) higher loneliness score than respondents who indicated they did not need support or those who indicated they did need support and received it. Male, Black, and married respondents were less lonely than their counterparts (female, White, unmarried respondents) when controlling for other factors. However, the coefficients for Black and married were no longer significant when controlling for pre-pandemic loneliness. This suggests that only female respondents were more likely than males to become lonelier after the start of the pandemic.

## 4. Discussion

The current study aimed to examine the role of social and built environment factors in shaping COVID-19 experiences of disruption and loneliness in a Gulf Coast sample. As such, the study aimed to help answer questions about how investment in different features of the social and built environment, considered together, could impact loneliness and other key health outcomes, such as mental health, within and beyond pandemics [10,19,21,77] (Astell-Burt et al. 2022; Bower, Kent, et al. 2023; Evans 2003; Meehan et al. 2023).

Overall, the results of this study suggest that respondents were still experiencing disruption due to COVID-19 in May 2022 and that they felt lonelier during the pandemic when compared to pre-pandemic times. Furthermore, higher disruption to routine behaviors due to COVID-19 was associated with experiencing increased loneliness. These results are generally in line with findings from a meta-analysis that found COVID-19 resulted in small yet heterogeneous increases in loneliness [27,29]. Results from regression testing associations between social and built environment factors and our outcomes revealed that difficulty in accessing green space and difficulties inside one’s household (e.g., lack of space) were associated with greater disruption. Housing distress was also associated with higher loneliness scores, but that effect was no longer significant once disruption was controlled for. This may suggest that separation between work and home and indoor and outdoor spaces allowing safe social interactions may have reduced loneliness. Furthermore, lower perceptions of neighborhood safety were associated with more loneliness during the pandemic, suggesting that safer neighborhoods could protect against loneliness. For example, residents of safe neighborhoods may have felt more comfortable spending extended periods of time outside and in public spaces where they could interact with other community members. Together, these results echo other studies that have demonstrated the importance of housing conditions, safe neighborhoods, and access to outdoor space in improving mental health and reducing loneliness during COVID-19 [45,78,79]. In our study, lacking support after experiencing impacts from hurricanes and storms between 2019–2022 also was associated with experiencing more loneliness over the course of the pandemic. This is consistent with other research finding community resilience to be an important predictor of loneliness in the Gulf Coast [80].

However, not all features of the social and built environment showed significant associations with both outcomes. While perceived difficulty in accessing green space was associated with experiencing more COVID-19 disruption, it was not associated with loneliness. As greater COVID-19 disruption was associated with loneliness, those results may be suggestive of a partially mediated relationship. Neighborhood social cohesion was not associated with loneliness, but this is due to collinearity:. Neighborhood social cohesion was highly correlated with neighborhood safety (r = 0.48, *p* < 0.001), which itself was associated with loneliness. In terms of walkability, there was little evidence suggesting that it mitigated feelings of loneliness despite prior studies suggesting it may be a protective factor [32,81,82,83]. However, there maybe a few explanations for this finding. Cronbach’s alpha for the scale measuring perceptions of neighborhood walkability indicated poor reliability (0.43), suggesting the scale may not have reliably captured the construct, especially since this scale was also not correlated with the walkability index at the census block group level. This suggests that the subjective scale and the walkability index may have captured different elements related to walkability (traffic speed vs. aesthetics and land use) since previous studies found objective measures to be predictive of walking outcomes [46]. While we did not find evidence for the hypothesis that walkable neighborhoods reduce loneliness, it is worth considering if this could also be explained by the “uncertain geographic context problem [21,84]. The issue here would suggest that the census block group may not be the “true” spatial configuration where walkability characteristics have a significant impact on loneliness. Census block groups may be too small (or too large) of a geographic scale to capture walkability in a manner relevant to facilitating individual or collective mobility that fosters opportunities and access to social connection. Future research should explore walkability at different levels (smaller or block level and larger or tract level) and consider more specific mechanisms that explain how walkability impacts loneliness in a way that can inform an appropriate geographic context.

An interesting finding was that objective neighborhood characteristics were not strongly associated with COVID-19 disruption or loneliness when accounting for subjective measures of the social and built environment. This echoes several studies that found mixed results when considering associations between objective measures, subjective measures, and related health outcomes [19,85,86]. This may be because subjective accessibility is more important for outcomes like loneliness than the availability of a physical structure or the decontextualized design of a place. In this study, the difficulty in accessing green areas was more salient to experiencing disruption and loneliness than the number of parks in a neighborhood. This is consistent with the view that loneliness is a subjective experience determined through the interaction of individual expectations and abilities, a community’s built environment, and socio-structural factors [87]. In that sense, an environment is only protective against loneliness in as much as it allows connection and belonging, which depends on the context of the individual situated in the physical place [21].

## 5. Limitations

These results should be considered in the light of a few limitations. The study’s moderate sample size and significant attrition from the original cohort in the first wave of STRONG might limit the generalizability of results to the Gulf Coast region. We attempted to address this by noting that our sample did not significantly differ from the original cohort in the first wave on main sociodemographic factors (see Table A1 in Appendix A). Still, the findings in this study may not generalize to younger individuals, as the sample (in both waves) was skewed towards older adults when compared to the 5-year estimates from the 2016 American Community Survey [61]. We also cannot rule out that attrition between the first wave and the third wave was related to some other unmeasured characteristics. This would especially bias our estimates if those characteristics are related to the outcomes in the study (COVID-19 disruption and loneliness).

Additionally, the study’s cross-sectional design prevents strict causal inferences, with potential biases due to omitted variables. It is possible there are other variables (e.g., health or personality) that may affect both the responses to subjective social–built environment items and our outcomes. We also cannot rule out reverse causality between loneliness, COVID-19 disruption, and environmental factors. For example, susceptibility to loneliness could influence where people decide to live, which would mean aspects of the social and built environment are not strictly exogenous factors. While we attempted to account for some of this exogeneity in predicting loneliness during the pandemic by controlling for a pre-pandemic loneliness measure (Model 8), several studies have shown that this specification can lead to regression artifacts. These artifacts can bias estimates in non-experimental settings where the predictor is correlated with the outcome measured at baseline or where the outcome measured at baseline exhibits measurement error [88,89,90].

Finally, a few of the measures used were imperfect. The neighborhood walkability measure had low reliability, which may challenge the finding that neighborhood walkability did not matter for COVID-19 disruption and loneliness experiences. The use of a retrospective pre-pandemic loneliness measure also introduces complications, including potential recall bias and correlation with predictors, which may overestimate some associations and could be influenced by how public discourse on loneliness has evolved throughout the pandemic. For this reason, we present models both including and excluding pre-pandemic loneliness as a predictor.

## 6. Conclusions

This study explored the interplay between the social and built environments and their associations with routine disruption and loneliness among residents of the U.S. Gulf Coast during the COVID-19 pandemic. The findings illuminate the significant role that subjective measures of the social and built environment may play in shaping experiences of loneliness and disruption caused by the pandemic. Particularly, limited access to green spaces was associated with COVID-19 disruption. Conversely, perceived neighborhood safety and housing security appeared to be protective factors against loneliness, buffering residents from the psychological impacts of the pandemic. These findings underscore the potential benefits of interventions that consider both the physical and social fabrics of communities. They also highlight the importance of urban planning and community support mechanisms, especially in preparing for future public health crises. Specifically, policymakers should consider enhancing the safety of neighborhoods and fostering community cohesion as strategies to mitigate loneliness and adverse mental health outcomes among adults. The results of this study highlight that access to green spaces rather than mere proximity is a significant predictor of disruption and loneliness. Therefore, urban planning policies should prioritize not only the provision of green spaces but also the enhancement of access to these areas. This can be achieved by carefully integrating green infrastructure into community design and improving pathways, links, and other methods of access. Additionally, design considerations must accommodate individuals with physical disabilities, special needs, and older adults to ensure equitable access. The relationship identified between a lack of social support following disasters and increased loneliness is particularly significant for the U.S. Gulf Coast, a region where hurricane risk is projected to escalate due to climate change [91]. These findings would suggest a critical role for community-based disaster risk management strategies. Policies that support local community-based disaster risk management are essential not only for enhancing adaptive capacity in the face of escalating environmental risks but also for improving mental health and reducing loneliness in a region prone to both environmental and health-related disruptions.

Looking forward, further research should explore the longitudinal impacts of environmental and other social-ecological factors on loneliness and other mental health outcomes beyond the pandemic. Studies involving a wider range of geographic locations could provide a more generalized understanding of these dynamics. Moreover, a deeper investigation into the role of individual differences in susceptibility to environmental influences on loneliness could offer personalized strategies to combat isolation among vulnerable populations.

## Figures and Tables

**Table 1:** Sample Demographics.

Variable	n (%), Before Multiple Imputation (N = 532)	%, With Multiple Imputation
**Age**		
18–29	14 (2.7%)	2.7%
30–45	52 (9.9%)	10.0%
46–64	162 (31%)	31%
65+	296 (56%)	56%
Missing	8	
**Female**	322 (61%)	61%
Missing	1	
**Hispanic**	41 (7.8%)	8.1%
Missing	4	
**Race**		
White	426 (80%)	80%
Black	93 (18%)	17%
Other	12 (2.3%)	2.3%
Missing	1	
**Married**	296 (56%)	56%
Missing	3	
**Living Alone**	23 (4.4%)	4.6%
Missing	7	
**Homeowner**	422 (81%)	81%
Missing	9	
**Employed**	203 (39%)	39%
Missing	9	
**Income (USD)**		
Less than 30,000	149 (30%)	30%
30,000–49,999	88 (18%)	18%
50,000–74,999	79 (16%)	16%
75,000– or more	176 (36%)	36%
Missing	40	

**Table 2:** Distributions of study outcomes and main predictors.

Variable	Mean ± SD (Range) or %, Original Sample [N = 532]	Mean ± SD (Range) or %, With Multiple Imputation
**Outcomes**		
Pre-pandemic Loneliness	0.86 ± 1.41 (0.00, 6.00)	0.87 ± 1.42 (0.00, 6.00)
Loneliness During the Pandemic	2.09 ± 2.03 (0.00, 6.00)	2.09 ± 2.03 (0.00, 6.00)
Disruption to Routine Behaviors	3.7 ± 4.8 (0.0, 24.0)	3.8 ± 4.8 (0.0, 24.0)
**Social and Built Environment (Objective)**		
Number of Parks in Neighborhood (Census Tract)	1.56 ± 2.04 (0.00, 18.00)	1.56 ± 2.04 (0.00, 18.00)
Walkability Index (Census Block Group)	8.1 ± 3.5 (1.7, 18.8)	8.1 ± 3.5 (1.7, 18.8)
Urban (Census Tract)	90%	90%
Percent of Neighborhood Living in Poverty (Census Tract)	14 ± 11 (0, 61)	14 ± 11 (0, 61)
**Social and Built Environment (Subjective)**		
Neighborhood Social Cohesion	13.5 ± 3.7 (0.0, 20.0)	13.7 ± 3.7 (0.0, 20.0)
Neighborhood Safety	12.0 ± 3.3 (0.0, 16.0)	12.0 ± 3.3 (0.0, 16.0)
Access to Green Space	2.77 ± 1.09 (0.00, 4.00)	2.77 ± 1.09 (0.00, 4.00)
Neighborhood Walkability	7.26 ± 2.31 (0.00, 12.00)	7.28 ± 2.31 (0.00, 12.00)
Housing Distress	1.21 ± 1.91 (0.00, 14.00)	1.22 ± 1.91 (0.00, 14.00)
Lacked Needed Support After 2019–2022 Storms	18%	18%

**Table 3:** Bivariate associations between loneliness, disruption, lacking post-disaster support, and characteristics of the social and built environment.

Variable	1	2	3	4	5	6	7	8	9	10	11	12
1. Pre-pandemic Loneliness												
2. Loneliness During the Pandemic	0.50 ***											
3. Disruption	0.28 ***	0.40 ***										
Subjective Social and Built Environment Measures												
4. Neighborhood Social Cohesion	−0.22 ***	−0.20 ***	−0.19 ***									
5. Neighborhood Safety	−0.16 ***	−0.24 ***	−0.16 ***	0.48 ***								
6. Access to Green Space	−0.11 **	−0.17 ***	−0.10 *	0.34 ***	0.31 ***							
7. Neighborhood Walkability	−0.14 **	−0.16 ***	−0.17 ***	0.19 ***	0.26 ***	0.34 ***						
8. Housing Distress	0.21 ***	0.20 ***	0.26 ***	−0.28 ***	−0.27 ***	−0.12 **	−0.06					
9. Lacked Needed Support	0.18 ***	0.21 ***	0.13 **	−0.19 ***	−0.22 ***	−0.18 ***	−0.18 ***	0.17 ***				
Objective Social and Built Environment Measures												
10. Count of Parks	−0.05	0.02	0.05	0.01	0.00	0.03	0.11 **	0.06	−0.08			
11. Neighborhood Poverty Rate	0.10 *	0.06	0.10 *	−0.04	−0.26 ***	−0.06	−0.05	0.18 ***	0.07	−0.01		
12. Urban Tract	−0.08	−0.02	0.00	−0.02	−0.08	−0.05	−0.01	0.00	0.01	−0.06	−0.02	
13. Walkability Index	0.10 *	0.10 *	0.04	−0.04	−0.22 ***	0.02	0.02	0.12 **	0.03	0.20 ***	0.17 ***	0.12 **

Note: *** *p* < 0.001, ** *p* < 0.01, and * *p* ≤ 0.05; measure of association is Pearson correlation.

**Table 4:** Multiple Linear Regression Predicting Disruption to Routine Behaviors.

Variables	Model 1	Model 2	Model 3
	Beta (95% CI)	Beta (95% CI)	Beta (95% CI)
**Objective Social and Built Environment Measures**			
Number of Parks in Neighborhood (Census Tract)	0.11 (−0.09, 0.32)	0.14 (−0.06, 0.34)	0.17 (−0.02, 0.37)
Walkability Index (Census Block Group)	0.02 (−0.10, 0.14)	−0.01 (−0.13, 0.11)	−0.06 (−0.18, 0.06)
Percent of Neighborhood Living in Poverty (Census Tract)	0.04 (0.01, 0.08) *	0.02 (−0.02, 0.06)	−0.03 (−0.07, 0.02)
Urban (Census Tract)	0.12 (−1.2, 1.5)	0.10 (−1.2, 1.4)	0.07 (−1.2, 1.4)
**Subjective Social and Built Environment Measures**			
Neighborhood Social Cohesion		−0.12 (−0.25, 0.00)	−0.13 (−0.26, 0.00) *
Neighborhood Safety		−0.01 (−0.16, 0.14)	0.02 (−0.13, 0.17)
Access to Green Space		−0.60 (−1.0, −0.21) **	−0.53 (−0.91, −0.14) **
Neighborhood Walkability		0.03 (−0.16, 0.22)	0.00 (−0.19, 0.19)
Housing Distress		0.51 (0.29, 0.73) ***	0.39 (0.17, 0.61) ***
Lacked Needed Support After 2019–2022 Storms		0.73 (−0.34, 1.8)	0.18 (−0.89, 1.2)
**Demographic Covariates**			
Age			
18–29			—
30–45			−0.24 (−3.1, 2.6)
46–64			−0.40 (−3.0, 2.2)
65+			−0.95 (−3.6, 1.7)
Female			0.44 (−0.40, 1.3)
Hispanic			1.8 (0.34, 3.3) *
Race			
White			—
Black			2.4 (1.3, 3.6) ***
Other			−0.52 (−3.2, 2.1)
Married			−0.91 (−1.8, 0.01)
Living Alone			−1.9 (−3.9, 0.15)
Homeowner			0.41 (−0.66, 1.5)
Employed			−0.97 (−2.0, 0.09)
Income (USD)			
Less than 30,000			—
30,000–49,999			−0.01 (−1.2, 1.2)
50,000–74,999			−1.2 (−2.5, 0.20)
75,000–or more			−0.46 (−1.7, 0.79)

Note: *** *p* < 0.001; ** *p* < 0.01; * *p* < 0.05.

**Table 5:** Multiple Linear Regression Predicting Loneliness During the COVID-19 Pandemic.

Variables	Model 4	Model 5	Model 6	Model 7	Model 8
	Beta (95% CI)	Beta (95% CI)	Beta (95% CI)	Beta (95% CI)	Beta (95% CI)
**Objective Social and Built Environment Measures**					
Number of Parks in Neighborhood (Census Tract)	0.00 (−0.08, 0.09)	0.02 (−0.06, 0.11)	0.00 (−0.08, 0.08)	−0.01 (−0.08, 0.07)	0.02 (−0.05, 0.10)
Walkability Index (Census Block Group)	0.06 (0.01, 0.11) *	0.04 (−0.01, 0.09)	0.04 (−0.01, 0.09)	0.03 (−0.01, 0.08)	0.02 (−0.03, 0.06)
Percent of Neighborhood Living in Poverty (Census Tract)	0.01 (−0.01, 0.02)	0.00 (−0.02, 0.01)	−0.01 (−0.02, 0.01)	−0.01 (−0.03, 0.01)	−0.01 (−0.02, 0.01)
Urban (Census Tract)	−0.19 (−0.77, 0.38)	−0.27 (−0.82, 0.28)	−0.29 (−0.80, 0.23)	−0.24 (−0.75, 0.28)	0.01 (−0.46, 0.49)
**Subjective Social and Built Environment Measures**					
Neighborhood Social Cohesion		−0.03 (−0.08, 0.02)	−0.01 (−0.06, 0.04)	−0.01 (−0.07, 0.04)	0.01 (−0.04, 0.05)
Neighborhood Safety		−0.07 (−0.13, −0.01) *	−0.07 (−0.13, −0.01) *	−0.06 (−0.12, 0.00)	−0.06 (−0.12, −0.01) *
Access to Green Space		−0.15 (−0.31, 0.02)	−0.06 (−0.22, 0.09)	−0.03 (−0.18, 0.13)	0.00 (−0.14, 0.14)
Neighborhood Walkability		−0.05 (−0.13, 0.03)	−0.05 (−0.13, 0.02)	−0.05 (−0.12, 0.03)	−0.04 (−0.11, 0.03)
Housing Distress		0.13 (0.03, 0.22 )**	0.05 (−0.04, 0.14)	0.04 (−0.05, 0.13)	0.01 (−0.07, 0.09)
Lacked Needed Support After 2019–2022 Storms		0.68 (0.23, 1.1) **	0.57 (0.15, 1.0) **	0.57 (0.15, 0.99) **	0.41 (0.02, 0.79) *
Disruption to Routine Behaviors			0.14 (0.11, 0.18) ***	0.14 (0.10, 0.17) ***	0.11 (0.07, 0.14) ***
Pre-pandemic Loneliness					0.55 (0.44, 0.66) ***
**Demographic Covariates**					
Age					
18–29				—	—
30–45				−0.02 (−1.1, 1.1)	−0.01 (−1.0, 0.99)
46–64				−0.39 (−1.4, 0.62)	−0.18 (−1.1, 0.76)
65+				−0.30 (−1.3, 0.72)	−0.03 (−0.98, 0.92)
Female				0.60 (0.27, 0.93) ***	0.64 (0.34, 0.95) ***
Hispanic				−0.06 (−0.66, 0.54)	0.07 (−0.48, 0.63)
Race					
White				—	—
Black				−0.60 (−1.1, −0.13) *	−0.42 (−0.85, 0.02)
Other				−0.84 (−1.9, 0.21)	−0.62 (−1.6, 0.35)
Married				−0.61 (−0.97, −0.24) **	−0.24 (−0.59, 0.10)
Living Alone				−0.06 (−0.84, 0.72)	0.02 (−0.72, 0.75)
Homeowner				−0.02 (−0.44, 0.40)	0.14 (−0.25, 0.52)
Employed				0.06 (−0.36, 0.47)	0.18 (−0.20, 0.56)
Income (USD)					
Less than 30,000				—	—
30,000–49,999				0.32 (−0.18, 0.81)	0.43 (−0.03, 0.89)
50,000–74,999				0.06 (−0.48, 0.59)	0.23 (−0.27, 0.73)
75,000–or more				−0.16 (−0.66, 0.33)	−0.02 (−0.48, 0.44)

Note: *** *p* < 0.001; ** *p* < 0.01; * *p* < 0.05.

## Data Availability

The data that support the findings (except for geographic identifiers) will be available in the Gulf Science Data Repository (GRIIDC at https://data.griidc.org/, accessed on 26 January 2025) following an embargo from the conclusion of the project.

## Appendix A

**Table A1:** Table comparing the original sample that participated in Wave 1 to the study sample (participated in Wave 3) on main sociodemographic characteristics.

Respondent Characteristics	n(%), Wave 1 Respondents (N = 2520)	n(%), Wave 3 Respondents (N = 598)	%, 2016 ACS (5-Year Estimates)
**Sex**			
Male	994 (40%)	224 (37%)	48.7%
Female	1522 (60%)	374 (63%)	51.3%
**Age Group**			
18–24	154 (6.1%)	26 (4.3%)	11.7%
25–34	207 (8.2%)	35 (5.9%)	17.9%
35–44	245 (9.7%)	47 (7.9%)	16.4%
45–54	395 (16%)	110 (18%)	17.2%
55–64	565 (22%)	162 (27%)	16.2%
65+	950 (38%)	218 (36%)	20.5%
**Hispanic**	262 (10%)	47 (7.9%)	22.6%
**Race**			
White	1917 (76%)	478 (80%)	78.2%
Black	530 (21%)	106 (18%)	15.9%
Asian	29 (1.2%)	5 (0.8%)	3.7%
American Indian or Native	34 (1.4%)	9 (1.5%)	0.45%
Native Hawaiian or Pacific	6 (0.2%)	0 (0%)	0.05%
**Marital Status**			
Married	1205 (48%)	330 (55%)	-
Widowed	429 (17%)	74 (12%)	-
Divorced	374 (15%)	88 (15%)	-
Separated	88 (3.5%)	22 (3.7%)	-
Never married	420 (17%)	84 (14%)	-
**Education**			
<HS	199 (7.9%)	23 (3.8%)	15.7%
HS	618 (25%)	137 (23%)	28.6%
Some College	518 (21%)	127 (21%)	23.3%
Assoc/Voc	348 (14%)	75 (13%)	7.6%
Bachelors	468 (19%)	128 (21%)	16.2%
Graduate	365 (15%)	108 (18%)	8.7%
**Household Income (USD)**			
Less than 10,000	201 (8.0%)	40 (6.7%)	7.41%
10,000–49,999	1236 (49%)	266 (45%)	41.60%
50,000–74,999	374 (15%)	96 (16%)	17.90%
75,000–99,999	318 (13%)	86 (14%)	11.36%
100,000 or more	387 (15%)	110 (18%)	21.71%
